# Neurofibromatosis Type 1: Clinical and Imaging Perspectives From a Pediatric Case

**DOI:** 10.1155/crra/9912392

**Published:** 2025-06-03

**Authors:** Puneet Kumar Choudhary, Ankit Kumar Meena, Arvinder Wander, Aakash Mahesan, Paramdeep Singh

**Affiliations:** ^1^Department of Pediatrics, All India Institute of Medical Sciences, Patna, India; ^2^Child Neurology Division, Department of Paediatrics, All India Institute of Medical Sciences, Bathinda, Punjab, India; ^3^Department of Pediatrics, All India Institute of Medical Sciences, New Delhi, India; ^4^Department of Radiodiagnosis, All India Institute of Medical Sciences, Bathinda, Punjab, India

**Keywords:** café-au-lait macules, focal areas of signal intensity (FASIs), Lisch nodules, neurofibromatosis

## Abstract

Neurofibromatosis (NF) is a common disorder that affects the nerves and skin. There are two main types: neurofibromatosis Type 1 (NF-1) (also called von Recklinghausen's disease) and neurofibromatosis Type 2 (NF-2) (previously known as bilateral acoustic NF or central NF). NF-1 makes up approximately 85% of cases, with a prevalence of 1 in 5000 in the general population. In 30%–50% of NF-1 cases, there is no family history, suggesting that these cases likely result from germ cell mutations, often from the father. Here, we present the case of a 7-year-old boy with skin and radiological features of NF-1. NF-1 is the most common neurocutaneous syndrome, requiring long-term monitoring for related complications. In this case, we aimed to highlight the typical clinical and radiological features of NF-1 in a child.

## 1. Introduction

Neurofibromatosis (NF) is a prevalent disorder characterized by neurological and cutaneous lesions. It manifests in two primary forms: neurofibromatosis Type 1 (NF-1) (also known as von Recklinghausen's disease) and neurofibromatosis Type 2 (NF-2) (previously identified as bilateral acoustic NF or central NF). NF-1 constitutes approximately 85% of cases, with a population prevalence of 1 in 5000. In 30%–50% of NF-1 cases, there is no family history, suggesting that these cases likely result from germ cell mutations, usually from the father. We present a case of a 7-year-old boy who presented with cutaneous and radiological features of NF-1.

## 2. Case Description

**Figure 1:**
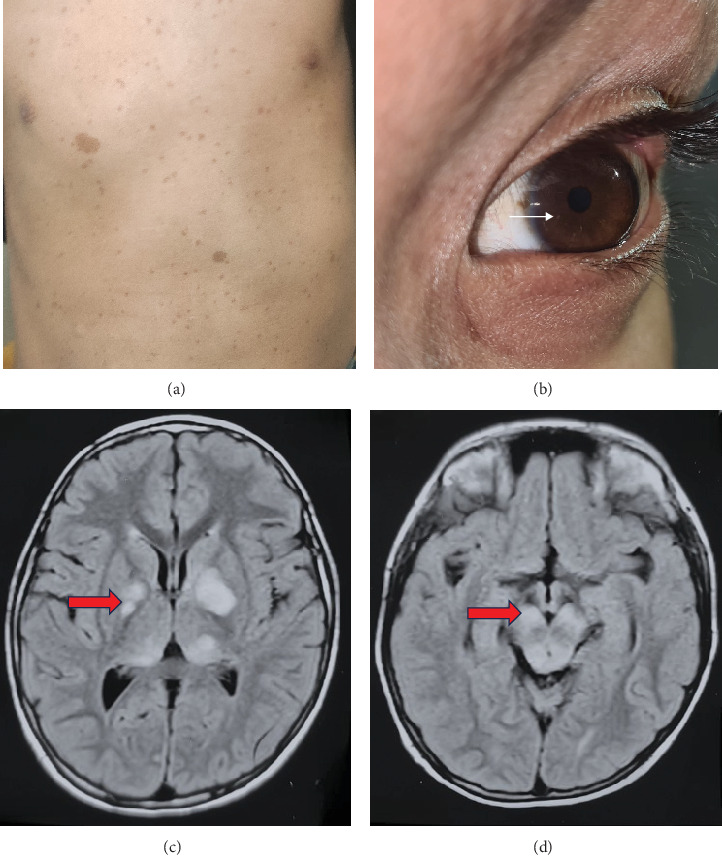
Clinicoradiological features of NF-1. Café-au-lait spots over the (a) chest and abdomen and (b) Lisch nodules seen over the iris. The MRI brain FLAIR axial section shows the focal areas of signal intensity (FASIs) indicated by red arrows in the (c) basal ganglia and thalamus and in the (d) brainstem.

A 7-year-old boy with normal developmental milestones presented with a painless, slow-growing soft swelling in the occipital region. He was referred to the pediatric outpatient department from the surgical unit for further evaluation of the occipital swelling. Clinical examination revealed the presence of multiple café-au-lait lesions and Lisch nodules in the eyes (Figure 1). His cognitive function was within the normal range, with good scholastic performance, and there was no history of seizures or behavioural issues. Lisch nodules were confirmed on slit-lamp examination. His brain MRI revealed hyperintense lesions on T2/FLAIR-weighted images in the basal ganglia, thalamus, and brainstem, which were consistent with focal areas of signal intensity (FASIs) found in NF-1 (Figure 1).

## 3. Discussion

NF-1 is an autosomal dominant neurocutaneous disorder categorized as phacomatoses, primarily stemming from mutations in the NF-1 tumor suppressor gene. This condition encompasses a broad spectrum of systemic manifestations, including multiple café-au-lait macules, intertriginous freckling, multiple cutaneous neurofibromas, subcutaneous or deep nodular neurofibromas, plexiform neurofibromas, and distinctive ocular findings [1].

The diagnostic criteria for NF-1 were originally established in 1987. In 2021, these criteria were revised to incorporate advancements in genetics, ophthalmology, dermatology, and neuroimaging, aiming to reduce diagnostic delay and increase diagnostic accuracy [2]. The key updates to the 2021 criteria include the following:

1. Inclusion of genetic testing: A pathogenic NF-1 gene variant identified in normal tissue now serves as a diagnostic criterion, enhancing diagnostic precision, especially in ambiguous cases.
2. Ophthalmologic findings: The presence of two or more choroidal abnormalities, detectable via optical coherence tomography (OCT) or near-infrared reflectance (NIR) imaging, has been added as a diagnostic feature, alongside the traditional criterion of two or more Lisch nodules.
3. Osseous lesion specification: The revised criteria specify distinctive osseous lesions, including sphenoid dysplasia, anterolateral bowing of the tibia, or pseudarthrosis of a long bone, providing clearer guidelines for diagnosis.
4. Clarification of the pigmentary findings: While café-au-lait macules and freckling remain diagnostic features, the updated criteria recommend that at least one of these pigmentary findings be bilateral to improve diagnostic accuracy.
5. Consideration of family history: A child with a parent who meets the NF-1 diagnostic criteria can be diagnosed with NF-1 if one or more of the specified criteria are present in the child, emphasizing the importance of family history in diagnosis.

These revisions were aimed at increasing the accuracy and timeliness of NF-1 diagnoses, facilitating improved patient care and management.

Café-au-lait macules are flat, uniformly hyperpigmented skin lesions that typically manifest within the first year of life and often proliferate during early childhood. Notably, up to 15% of the general population may have one to three café-au-lait macules; nevertheless, the presence of six or more of these macules strongly raises suspicion for NF-1 [3]. Lisch nodules are elevated, tan-coloured hamartomas on the iris that constitute a distinctive indicator of NF-1. Notably, these nodules are observed in fewer than 10% of children with NF-1 who are under the age of 6, yet they are prevalent in more than 90% of adult individuals with NF-1. Thus, their presence serves a dual purpose in the context of an NF-1 diagnosis: aiding in confirming the condition of a child and assisting in determining whether a parent is affected by NF-1 [4]. It is important to examine the eyes carefully to look for Lisch nodules when NF-1 is suspected and to confirm them with a slit-lamp examination. FASIs are frequent findings in children with NF-1, but they may tend to diminish or even disappear as individuals reach adulthood. These bright spots are typically located in the basal ganglia, cerebellum, brainstem, and subcortical white matter. They are believed to be associated with an increase in fluid within the myelin space due to dysplastic glial proliferation. Importantly, these bright spots are not included in the consensus diagnostic criteria for NF-1.

Consensus guidelines from the American Academy of Pediatrics and the American College of Medical Genetics recommend annual examinations for children with NF. During these visits, the child should be evaluated for the following: the presence of new fibromas or plexiform neuromas, blood pressure monitoring, growth assessment, skeletal changes, a formal ophthalmological examination (including visual screening), the assessment of precocious puberty, and a neurodevelopmental evaluation. These evaluations are essential for the early detection and management of potential complications associated with NF.

The management of NF-1 requires a multidisciplinary approach, as there are changes in the diagnostic criteria, and advancements in management have also taken place. Selumetinib, an oral selective mitogen-activated protein kinase kinase (MEK) inhibitor capable of inducing tumor regression, was approved by the US Food and Drug Administration (FDA) in April 2020 for the treatment of pediatric patients aged 3 years or older with symptomatic and/or progressive, inoperable NF-1–related plexiform neurofibromas.

## 4. Conclusion

NF-1 is the most common neurocutaneous syndrome and needs long-term surveillance for its related complications. In this case, we have attempted to present the classical clinical–radiological features of NF-1 in a child.

## Data Availability

The data that support the findings of this case report are available from the corresponding author upon reasonable request.
